# Gattaca: Base-Pair Resolution Mutation Tracking for Somatic Evolution Studies using Agent-based Models

**DOI:** 10.1093/molbev/msac058

**Published:** 2022-03-17

**Authors:** Ryan O. Schenck, Gabriel Brosula, Jeffrey West, Simon Leedham, Darryl Shibata, Alexander R.A. Anderson

**Affiliations:** 1 Integrated Mathematical Oncology, H. Lee Moffitt Cancer Center & Research Institute, Tampa, FL 33612, USA; 2 Wellcome Centre for Human Genetics, University of Oxford, Oxford OX37BN, United Kingdom; 3 Department of Pathology, University of Southern California, Keck School of Medicine, Los Angeles, CA 90033, USA

**Keywords:** somatic evolution, computational tool, mechanistic modeling

## Abstract

Research over the past two decades has made substantial inroads into our understanding of somatic mutations. Recently, these studies have focused on understanding their presence in homeostatic tissue. In parallel, agent-based mechanistic models have emerged as an important tool for understanding somatic mutation in tissue; yet no common methodology currently exists to provide base-pair resolution data for these models. Here, we present Gattaca as the first method for introducing and tracking somatic mutations at the base-pair resolution within agent-based models that typically lack nuclei. With nuclei that incorporate human reference genomes, mutational context, and sequence coverage/error information, Gattaca is able to realistically evolve sequence data, facilitating comparisons between in silico cell tissue modeling with experimental human somatic mutation data. This user-friendly method, incorporated into each in silico cell, allows us to fully capture somatic mutation spectra and evolution.

## Introduction

Recent studies examining histologically normal human and murine tissue have shown that a surprising admixture of somatic mutations can exist and even expand to a significant clonal area (Martincorena et al. 2015; Simons 2016a; Martincorena et al. 2018; Lee-Six et al. 2018; Colom et al. 2020). These studies have largely focused on mutation characterization and have had limited tools to offer explanations for the dynamics driving observed evolutionary trajectories, with only a few notable exceptions. Fewer still have begun incorporating agent-based models as a tool to explore somatic evolution in spatially constrained tissue (Sottoriva et al. 2015; Murai et al. 2018; Schenck et al. 2019; Colom et al. 2020). Historically, genomes within agent-based models have been represented as simple counters or as binary arrays. These binary arrays serve only as a proxy to then relate back to the genome of patients or animal models through heterogeneity measures and simple counts. Most notably, passenger/driver model genomes are simple counters that increase and decrease based on a set mutation rate and probability of driver acquisition. These are then tied to some function defined in the model, sometimes in very arbitrary ways. These studies have lacked the ability to compare, at base-pair resolution the mutation spectra, or utilize common tools designed for genotypical data (such as dN/dS and subclonal reconstruction tools designed for noisy biological data).

To date, there is not a tool that allows for somatic mutation induction and tracking at a base-pair resolution for mechanistic agent-based models. Previously, our understanding of mutational processes and sequencing technologies has not had the resolution or technical understanding necessary to create such a tool, not to mention the computational power needed for such a task. Due to substantial advances, this has quickly become possible. First was our improved understanding of mutational processes (Lawrence et al. 2013). Then, we began to grasp the importance of heterogeneity in tumors through multiregion sequencing studies (Gerlinger et al. 2012). Now, we have added to the wealth of research to understand mutation in normal, homeostatic tissues (Martincorena et al. 2015). Currently, there is an ongoing debate over neutral and non-neutral dynamics in both tumors and normal tissue (Martincorena et al. 2016; Simons 2016b; Williams et al. 2016; Balaparya and De 2018; Heide et al. 2018; McDonald et al. 2018; Tarabichi et al. 2018; Werner et al. 2018; Williams et al. 2018; Leroi et al. 2020). Lastly, only recently mechanistic models have begun to really intersect with bioinformatics (Sottoriva et al. 2015; Colom et al. 2020; West et al. 2021). Together, these have facilitated the need for new methodologies where realistic genetic information can be incorporated into mechanistic models for future researchers.

Gattaca is the first tool that provides a means of inducing and tracking base-pair resolution single nucleotide variants within an agent-based modeling framework with temporal, spatial, and genomic positional information. The primary goal of Gattaca is to provide an users with a means to perform “null” simulations that are capable of explicitly modeling neutral somatic mutation within spatial contexts; however, beyond this, users can adapt this methodology for non-neutral dynamics through careful introduction of functional heterogeneity. This crucially provides an ability to accurately capture mutation data on a level comparable to sequencing experiments from the clinic or research settings.

## New Approaches

**Fig. 1.**
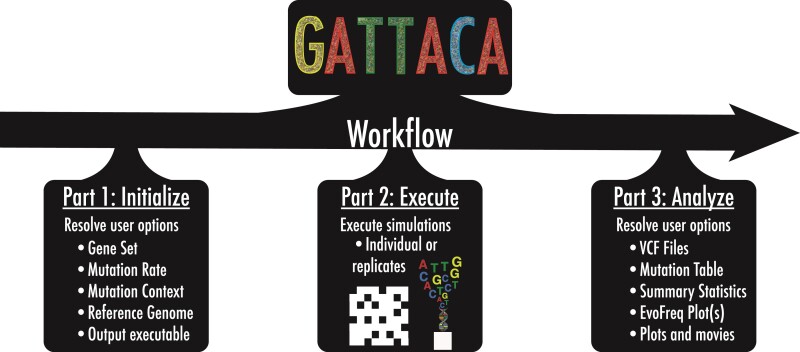
Gattaca is a three-part workflow for simulating base-pair resolution mutations within the human genome for somatic evolution in silico studies. Gattaca consists of three parts: *(i)* user defines options (initialize), *(ii)* generate a java executable class for in silico simulations with base-pair resolution mutation tracking (execution), and *(iii)* analyze the output of these simulations for downstream analysis (analyze).

Gattaca is provided as an easily executed python script consisting of three parts: initialization, execution, and analysis (fig. 1). After the initial set-up, Gattaca produces a java file that allows for integration into an agent-based model (ABM). The only pre-requisite for Gattaca is the installation of snpEff (Cingolani et al. 2012), which provides the necessary base-pair resolution reference genome and the tools to access it before Gattaca digests this information for downstream uses.

### Part 1: Initialization

The set-up resolves user inputs that includes mutation rates, mutation context probabilities, a gene set, and reference genome choice. Once resolved Gattaca extracts the gene locations from within the users’ reference genome; a Browser Extensible Data file is created that snpEff uses to extract bases for each gene. Gattaca then reads the provided mutational context file, in the event of none being provided a uniform probability is used. This file represents the probability of observing a given mutation from the 96 possible mutations within their trinucleotide contexts. Lastly, the mutation rates are scaled to the desired mean mutation rate. The mutation rates are adjusted from the gene-specific mutation rates derived from a pan-cancer study (Lawrence et al. 2013). This information is then prepared to generate a Gattaca java class tailored for execution within an ABM framework such as HAL (Bravo et al. 2020).

The heart of Gattaca is its ability to track mutations within simulations at a base-pair resolution. This requires a series of steps during each cell division where a user checks for mutation. The expected number of mutations per division is given for each gene ($g_{i}$) by the product of its individual mutation rate $\mu_{g_{i}}$ and its length $L_{g_{i}}$. Within each mutation check, during division a Poisson distribution is used to determine the number of mutations accrued for each gene ($X_{g}$), so that $X_{g}∼\mathrm{Poisson}(\mu_{g}\;*\;L_{g})$.

Determining the specific base that acquires a mutation is based on a multinomial of the 32 possible mutation positions based on trinucleotide contexts. This is drawn from a multinomial distribution based on the 32 possible positions. Once the trinucleotide is determined, the base mutation is determined using the mutation context probabilities to determine the mutation type.

### Part 2: Execution

Simulations utilizing Gattaca require the two files that are output by the Gattaca initialization step. These files, a java Gattaca class and a csv file with loci information, will be placed within the scope of your executable HAL model (Bravo et al. 2020). Details on using HAL can be found at http://halloworld.org. Once these are added to HAL, the Gattaca class will require initialization for a founding clone/population. Gattaca ties conveniently into the HAL phylogeny tracker requiring minimal additional computational overhead. Once Gattaca is initialized, a function call to *_RunPossibleMutation* will be required during each division that will trigger the possibility of mutation upon division as outlined above. A detailed tutorial on integrating Gattaca and HAL can be found at https://github.com/MathOnco/Gattaca.

Out of the box, Gattaca provides its users with the necessary genetic underpinnings for a purely neutral simulation, for example, mutations convey no functional advantage to a cell with any particular mutation. Should the user be interested in introducing functional heterogeneity based on mutations, the user would have to predetermine which mutation(s) will result in a functional difference. If this is tied to a specific mutation, the user must simply check the Gattaca genome during simulations where the functional advantage would be conveyed for that cell. However, due to the stochastic nature of when and where in the genome the mutation will occur, it may be valuable to induce the mutation manually at a specific time to evaluate simulations with non-neutral dynamics. If a user wants to evaluate mutation number, say for a Muller’s ratchet study (Muller 1932, 1964), they would simply check the number of acquired mutations for a cell and define its fitness advantage. However, mutation annotation is not completed until the Analysis section of Gattaca below, so any knowledge of mutation type or effect would have to be known a priori. Further, a user could tie a distribution of fitness effects using Gattaca as the underlying genome in the same way as the above two non-neutral examples. All these genome queries/changes can be completed for any cell and/or genome during the simulation by accessing the genome class, details on mid-simulation genome access are provided in the documentation.

### Part 3: Analysis

Once simulations are complete, Gattaca introduces the appropriate noise for each mutation type, one of two ways (adapted from Williams et al. 2016). The true variant allele frequency (assuming heterozygosity), ${\mathrm{VAF}}_{t}$, is given from ${\mathrm{VAF}}_{t}=N_{i}/2N_{e}$, where $N_{i}$ is the number of cells with a given mutation and $N_{e}$ is the population size. The user can provide a list of depths for mutations within an experimental cohort or define a single value *sequencing* depth. If the user sets a single value for depth (*d*), the number of reads calculated for the depth of a variant, $D_{i}$, is drawn from a Poisson distribution, which yields $D_{i}∼\mathrm{Poisson}(d)$. If a user provides a distribution of depths from an experimental cohort Gattaca determines the shape parameters ($k_{c}$ and $p_{c}$) defining a gamma distribution to obtain $D_{i}$ so that $D_{i}=\mathrm{Gamma}(k=k_{c},p=p_{c})$. The number of reads for a given variant ($f_{i}$) is finally determined by $f_{i}=B_{0}(n=D_{i},p={\mathrm{VAF}}_{t})$. By taking the sequenced VAF (${\mathrm{VAF}}_{s}=\;f_{i}/D_{i}$) and applying a threshold (typically 0.005–0.1 depending on sequencing depth) Gattaca yields mutations that are comparable to what may be observed from sequencing of tissue. Gattaca assumes that mutations are heterozygous and does not explicitly track copy number alterations (CNAs); however, the outputs of the Gattaca analysis pipeline provides users with the necessary numbers to examine the assumption of heterozygosity by *post hoc* analysis of mutation data.

Once the variants are called based on the corrected VAF, variants are annotated with snpEFF and mutational position information is obtained. The user can output this information as a mutational table for every desired timepoint and every replicate simulation. As an additional output option users can also export variants from their simulations as a variant call format (VCF) file. This option allows for easy use in several bioinformatics downstream tools. Lastly, the execution of the analysis component of Gattaca provides several summary statistics for evolutionary dynamics, such as 1/f (Williams et al. 2016), first incomplete moment (Butler and McDonald 1989; Simons 2016a), a Muller plot [using the EvoFreq (Gatenbee et al. 2019) package], and a crude dN/dS measurement. We note that a true dN/dS would be expected to be the same across all simulations unless the user implements functional heterogeneity within their simulations based on a single, or collection of, point mutations.

## Case Study 1: Dimensionality

Gattaca allows us to track base-pair resolution genomes across any agent-based modeling dimension. Recent interest by ourselves and others in understanding how spatial architecture may affect clonal dynamics and measurements of neutrality motivates our case study (Martincorena et al. 2015; Simons 2016a; Noble et al. 2019; Schenck et al. 2019; West et al. 2021).

**Fig. 2.**
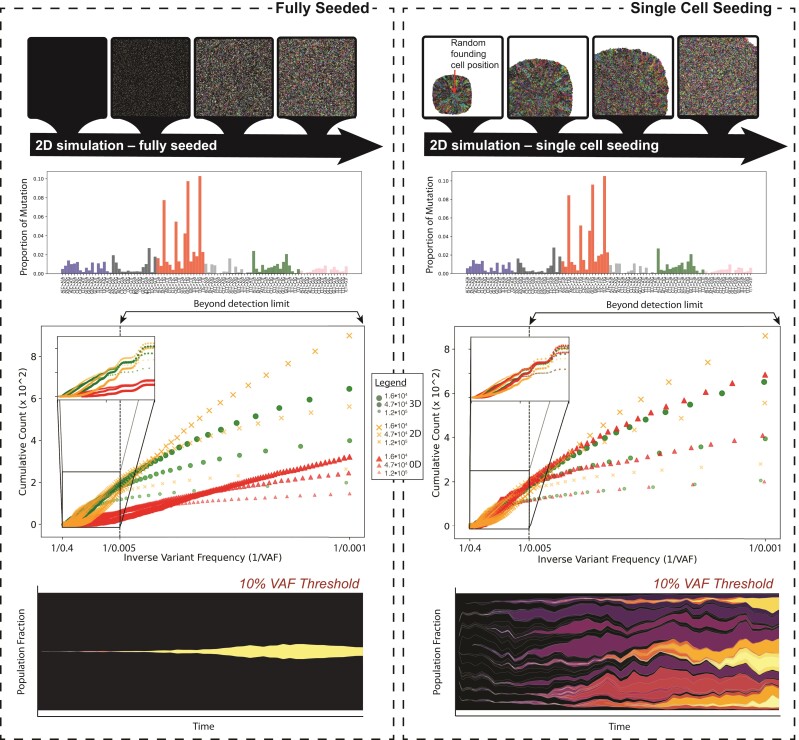
Results comparing the fully seeded (*left*) and single-cell seeding (*right*) model types and their corresponding mutation profiles and clonal dynamics. For each case, the mutation proportion across the 96 mutation trinucleotides is shown for one of the 2D simulation replicates. The 1/f values for each of the modeled dimensions are shown for three different population sizes, and the inset shows the 1/f distribution for that which would be within the limits of detection (a generous 0.005 VAF at high depths). Beneath this, the same 2D replicate that is shown in the mutation spectrum plot is used to highlight the differences in clonal dynamics using a Muller plot, produced using EvoFreq, with a 10% VAF cutoff between fully seeded and single-cell seeding.

Here, we have constructed two simple agent-based models (ABM) of cell turnover in three different dimensions, zero- (0D), two- (2D), and three-dimension (3D) to showcase and compare the mutational profiles and clonal dynamics that Gattaca allows its users to evaluate. In addition, we perform the simulations for these three dimensions and two model types for three different total final population sizes to demonstrate the functionalities and outputs of Gattaca (fig. 2). The two model types differ only in the number of cells that are present at initialization. The fully seeded model initializes by placing an agent with its unique genome at every lattice point or until the carrying capacity is reached in the 0D case. The second simple ABM is initialized only with a single cell at a random position within the simulated domain, or simply a population size of one for the 0D case (fig. 2). These two simple model types can be conceptualized as a naive tissue type of model to compare with a stem cell growth model similar to the idea that cancer originates from a single transformed clone. Here, we introduce no functional heterogeneity across the different genomes that emerge through mutation at each timepoint governed by the conditions set in Gattaca.

Within the two models, we use the same parameters so as to be able to more accurately compare across the different dimensions. Each model across all dimensions uses the same birth/death function. The birth rate (λ, λ=0.4) is scaled by the carrying capacity (*k*) and population size ($N_{T}$) at every time point of either the domain (e.g. number of lattice points) or as a set parameter in the 0D case. The equation governing this scaled birth rate ($\lambda_{T}$) is given by $\lambda_{T}=\lambda (\left(k-N_{T}\right)/k)$. If a random number ([0,1]) is less than the death parameter (ρ) plus $\lambda_{T}$, a death or birth may happen for a given cell. The probability of a birth event given an empty lattice position (2D and 3D only) is given by $P(\mathrm{Birth})=\rho +\lambda_{T}$. If a random number ([0,1]) is less than this birth event value a cell will die, if not the cell is able to divide.

When initializing Gattaca for these simulations, an overall mutation rate of $3.2\times 10^{-9}$ was used and the mutation spectrum defined was given from a randomly sampled cohort of diffuse large B-cell lymphoma whole exome sequences from TCGA (this is available in the gattaca example code). When we analyze these mutation spectrums, post simulation we observe similar distributions of mutation types across all dimensions and model types consistent with mutation processes expected, based on the Gattaca initialization (fig. 2 mutation spectrums). This cohort was chosen at random from the collection of TCGA sample types, but because of the way Gattaca uses this information to initialize distributions it is highly generalizable and any mutation spectra can be used. The differences that are observed largely depend on the dimensionality of the model chosen and the tissue type modeled. In the cases where the domain (or carrying capacity for 0D) is fully seeded, we see that the 1/f distributions of variant allele frequencies is similar in the 3D and 2D cases (fig. 2). Contrasting this with the single-cell seeding case we see that the 0D and 3D cases are the most similar while 2D appears to reveal a different distribution (fig. 2). These results suggest that the modeling dimension is an important consideration for the research question. As expected most of the clones that are observed are below the detection limits of common methodologies, but can be captured here. The clonal dynamics, as demonstrated by the EvoFreq plots (Gatenbee et al. 2019), illustrates that spatially constrained clones competing with one another are rarely able to expand beyond 10% VAF in the fully seeded cases while several clones reach this size during simulations with single-cell seeding.

## Case Study 2: Wounding

**Fig. 3.**
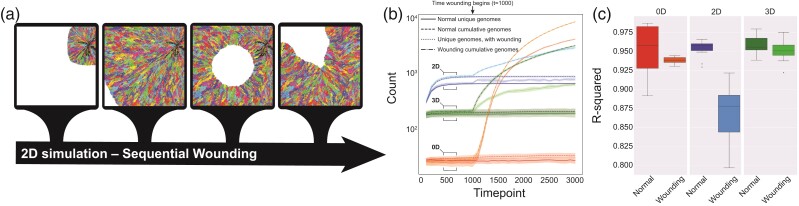
Illustration of the repeated wounding of the single-cell model in 2D where colors represent clones that differ by at least one mutation (*a*). For each of the dimensions, the cumulative and unique genomes is given over the course of simulations (*b*). *R*-squared values for the linear regression on 1/f distributions for mutations is plotted for all dimensions with and without wounding (*c*) for all replicate simulations.

Within the first case study, we utilized Gattaca across two different types of models and three different dimensions. Next, we wanted to evaluate if wounding within these models would alter the observed clonal dynamics as the spatial constraints for certain clones is relaxed when cells are removed in a wounding event (fig. 3*a*). In all simulations, each ABM is seeded by a single cell. Wounding begins once the thousandth timestep is reached (fig. 3*b*). After this, wounding occurs at time steps where the population is greater than or equal to 85% of the total possible population (as dictated by the domain size). For the 2D and 3D simulations, cells are killed by wounding within a circular and spherical manner, respectively. The number of cells killed through each wounding event is kept similar by adjusting the radius between 2D and 3D simulations, while in the 0D case, the number of cells killed is an equivalent number of cells. The same birth/death dynamics and equations used in case study one are used here, because the probability of birth is modulated by the number of cells (i.e. the probability of birth is reduced as the carrying capacity of the system is reached) a wounding event acts to increase cell divisions where empty sites are present and thus allows clones to expand into the wounded areas.

When we examine the differences between the wounding and non-wounding simulation’s cumulative and unique genomes over time, we see a clear signal at the time wounding occurs. At this point, space is open and rapid cell proliferation refills the areas where the wound occurred (in 0D this results in rapid proliferation back to carrying capacity). As cells divide and mutate, a large number of unique genomes appear over time (fig. 3*b*). We see that the number of unique genomes in the 0D case increases drastically faster than those in the 2D and 3D cases, this is due to the mechanism where clones in the 0D case are chosen at random to be killed while whole or near whole subclonal populations are removed in the 2D and 3D simulations. Interestingly, when we compare the 1/f distributions through their *R*-squared values, from linear regression analysis, we see that in the 2D wounding case, the relaxation of spatial constraints appears to drive a signal of non-neutral dynamics in a system that is functionally homogeneous where slight fitness advantages are conferred through room to expand (fig. 3*c*).

## Conclusions

Here we have presented Gattaca, the first base-pair resolution mutation induction and tracking in silico tool to model genomes within agent-based models. Gattaca provides a powerful tool to induce and track mutations through time and space to compare with patient and murine samples. Gattaca provides the necessary genomic underpinnings to evaluate “null” simulations that are capable of explicitly modeling neutral somatic mutation within spatial contexts that the user defines. We have demonstrated this by comparing the genomes and clonal dynamics that Gattaca provides across different modeling dimensions and model choices. We then show through a second use case that wounding can show evidence of selection, but only in the 2D wounding case. This sets an important precedent that modeling choices around dimensionality can significantly impact the measures of neutrality.

Gattaca at its heart is a simple tool built around users’ defined parameters that assumes mutations incurred are heterozygous, impart no fitness advantages to the cell, and can be easily deployed within most HAL simulations. This simplicity also allows for flexibility, in that users can evaluate CNAs post hoc with relative ease and introduce fitness advantages through careful consideration. Genotype–phenotype mapping is highly specific to an individual research question. If users decide to use Gattaca to introduce a genotype–phenotype mapping, great care should be taken to understand Gattaca’s limitations and the complexities/assumptions involved with introducing functional heterogeneity. Although Gattaca was built for and tested using the HAL agent-based modeling framework, with some effort, one could write a set of wrapper functions so that Gattaca could be used across different modeling frameworks.

Gattaca provides a highly customizable framework that is easily implemented into users’ agent-based simulations for evaluating somatic evolution in normal or disease tissue. Through the incorporation of common bioinformatics and genotypic outputs (variant call format files) used frequently in clinical and experimental approaches, users can quickly analyze and compare mutation spectra, burden, heterogeneity, and selection between their samples and in silico models.

## Supplementary Material

msac058_Supplementary_Data

## Data Availability

No new data has been generated here. Simulation outputs can be obtained through the examples in the code repository.

## Code Availability

Gattaca is available through GitHub (https://github.com/MathOnco/Gattaca). There is a read me available within the GitHub repository with further instructions on how to utilize Gattaca.
